# Effect of decomposition and organic residues on resistivity of copper films fabricated via low-temperature sintering of complex particle mixed dispersions

**DOI:** 10.1038/srep45150

**Published:** 2017-03-24

**Authors:** Yingqiong Yong, Mai Thanh Nguyen, Hiroki Tsukamoto, Masaki Matsubara, Ying-Chih Liao, Tetsu Yonezawa

**Affiliations:** 1 Division of Materials Science and Engineering, Faculty of Engineering, Hokkaido University, Kita 13 Nishi 8, Kita-ku, Sapporo, Hokkaido 060-8628, Japan; 2 Department of Materials and Environment Engineering, National Institute of Technology, Sendai College, 48 Nodayama, Medeshima-Shiote, Natori-shi, Miyagi 981-1239, Japan; 3 Department of Chemical Engineering, Faculty of Engineering, National Taiwan University, No. 1, Section 4, Roosevelt Rd., Da’an District, Taipei, 10617, Taiwan

## Abstract

Mixtures of a copper complex and copper fine particles as copper-based metal-organic decomposition (MOD) dispersions have been demonstrated to be effective for low-temperature sintering of conductive copper film. However, the copper particle size effect on decomposition process of the dispersion during heating and the effect of organic residues on the resistivity have not been studied. In this study, the decomposition process of dispersions containing mixtures of a copper complex and copper particles with various sizes was studied. The effect of organic residues on the resistivity was also studied using thermogravimetric analysis. In addition, the choice of copper salts in the copper complex was also discussed. In this work, a low-resistivity sintered copper film (7 × 10^−6^ Ω·m) at a temperature as low as 100 °C was achieved without using any reductive gas.

Over the past decades, direct printing of conductive inks/pastes has been emerging as an important technique to replace conventional photolithography, which is a complex, multiple-step method that releases a large amount of waste, for producing conductive films/patterns. In the printing technique, an ink or paste containing active materials is printed and then sintered to form conductive patterns or films. This makes the method cost-effective, highly efficient in terms of material usage, scalable and simple in terms of processing, and flexible for use on various types of substrates^1^,^2^,^3^. Many examples have been reported for using metallic inks in printed electronics, such as light-emitting diodes (LEDs)^4^, circuits^5^, flexible displays^6^, and radio frequency identification (RFID) tags^7^. Conductive inks or pastes based on silver are currently preferred because of their lowest resistivity (1.6 × 10^−8^ Ω·m) and stability in air^8^. However, these silver-based inks/pastes have two major disadvantages: high cost and low electro-migration resistance of silver. The former prevents the inks from meeting the low-cost requirement of the printing technique, while the latter can lead to circuit failure under high humidity conditions, thereby limiting their practical application on a large scale.

Copper nanoparticles and fine particles have been drawing extensive interest according to their potential applications^9^,^10^,^11^,^12^,^13^,^14^. Copper inks and pastes, which have a lower cost, excellent electrical resistivity (1.7 × 10^−8^ Ω·m which is similar to silver) and higher electro-migration resistance, have attracted significant interest^13^,^14^,^15^,^16^,^17^,^18^,^19^,^20^,^21^,^22^. Copper has a much lower price (approximately 2.6 USD/lb) than silver (approximately 17 USD/ounce)^23^. There are two types of copper inks being used^22^. One type is a suspension or dispersion of fine copper particles or nanoparticles^14^,^15^,^16^,^17^,^18^,^19^,^20^,^21^. The other type is the copper-based metal-organic decomposition (MOD) ink, which is composed of a copper salt, amine/amino hydroxyl ligands and other organic components^2^,^24^,^25^,^26^,^27^,^28^. Copper-based MOD ink, which has merits such as simplicity and scalability, has been studied for achieving high electrical conductivity at a low sintering temperature. Some additives, such as diethylene n-butanol^2^, glycolmethylether^2^, 1-methyl-2-pyrrolidinone^25^, octylamine^26^, and ethyl cellulose^25^, are generally required in the copper-based MOD inks for improving the uniformity of the prepared inks, increase conductivity and reliability of copper films after sintering, and for preventing liquid film dewetting or unwanted pinholes during the sintering process. However, these organic additives do not have good electrical conductivity and prevent contact between copper particles to some extent, thus decreasing the conductivity of the sintered copper films. In addition, to achieve low resistivity with such inks, high sintering temperature is needed. This hinders the use of low-cost electronic devices and flexible polymer substrate where lower sintering temperature is an essential factor.

In order to overcome these problems, we have proposed a new route: replacing the organic components with highly conductive commercial submicron copper particles, to improve the uniformity of the inks as well the conductivity of the sintered film at low sintering temperatures. In addition, we studied ligands of copper formate (CuF)/alkanolamines complexes^24^. In many cases, copper-based MOD ink was composed of CuF^2^,^24^,^25^,^26^,^27^,^28^ because CuF itself is self-reducible. Our results demonstrated that the added commercial copper particles, which directly contribute to the conductivity of the sintered films and to a higher packing density, resulted in the sintering of copper films at a low temperature, forming highly conductive copper films. A low resistivity of 9.0 × 10^−6^ Ω·m at a sintering temperature of 100 °C without a reductive gas was achieved. Further, for achieving a low resistivity (on the order of 10^−8^ Ω·m–10^−6^ Ω·m), mixing the copper complex with submicron copper particles for MOD inks offered a lower sintering temperature compared to mixing copper complex with other organic components^2^,^24^,^25^,^26^,^27^,^28^. This method is highly promising, but a detailed study of the copper salts for synthesizing copper complex and the effect of the size of the copper particles in MOD inks has not been reported so far.

In this work, besides CuF, copper acetate (CuA) was also used as the copper salt for the preparation of copper complex in the MOD inks, and the influence of copper sources on the conductivity of copper films was studied. As formic acid is a corrosive compound, acetic acid is more favourable for preventing failure of furnaces. The effect of the particle size of the copper particles in MOD inks on the resistivity of sintered copper films was investigated. In addition, the general applicability of this method was demonstrated using poly(vinylpyrrolidone) (PVP) capped copper particles as a substitute for commercial copper particles in MOD inks for obtaining highly conductive copper film.

## Experimental Section

### Preparation of commercial copper particle/CuA- (1-amino-2-propanol, IPA)-ethanol (EtOH) mixed inks (Cu/CuA-IPA-EtOH) and sintered copper films

Copper(II) acetate monohydrate (Cu(COOCH_3_)_2_·H_2_O, CuA) (Kanto, Japan) was used for the preparation of copper complex. Isopropanolamine (1-amino-2-propanol, IPA) (Junsei, Japan) was used as the ligand to form complexes with CuA. CuA (2.5 mmol, 0.50 g), IPA (5 mmol, 0.38 g), and ethanol (Kanto, 5 mmol, 0.23 g) were mixed using a conditioning mixer. Commercial copper fine particles without other oxidized copper species (50 wt%, Dowa, Japan, 1.11 g, median radius = 0.5 μm, Fig. S1) were added into the above mixture followed by mixing for 16 min with a conditioning mixer. The inks were deposited to a thickness of 40 μm on alumina substrates with a doctor blade, and were subsequently sintered at 120 °C and 150 °C under nitrogen gas for various durations in an electric tube furnace (As One). For a sintering at 120 °C, the heating speed was set at 2.3 °C min^−1^ from room temperature to 110 °C, followed by 1 °C min^−1^ to 120 °C. For a sintering at 150 °C, the heating speed was set at 2.3 °C min^−1^ from room temperature to 140 °C, then at 1 °C min^−1^ to 150 °C.

### Preparation of commercial copper particles/copper formate-IPA mixed inks (Cu/CuF-IPA) and sintered copper films

Copper(II) formate tetrahydrate (Cu(HCOO)_2_·4H_2_O, CuF, 5 mmol, 1.13 g) (Wako, Japan) and IPA (10 mmol, 0.75 g) were mixed well with a conditioning mixer. Commercial copper fine particles (Dowa, Japan) with a median radius of 0.4, 0.5, 0.8, and 2.5 μm (Fig. S1 and SI of reference paper^24^, the particle sizes were estimated by measuring size of at least 100 copper particles in SEM images) were mixed with a CuF-IPA complex using a conditioning mixer for 12 min. The copper fine particles were comprised 50 wt% of the ink. In addition, 70 wt% copper fine particles (0.4 μm) were also used to prepare the mixed ink. After mixing, the inks were printed to a thickness of 40 μm on alumina substrates using a doctor blade as above and dried under nitrogen for 1 h at 60 °C. Then, the printed inks were sintered at 120 °C or 100 °C under nitrogen gas for 30 min or 1 h in an electric tube furnace (As One). For a sintering at 120 °C, the heating speed was set at 2.3 °C min^−1^ from room temperature to 110 °C, then 1 °C min^−1^ from 110 °C to 120 °C. For a sintering at 100 °C, the heating speed was set at 2.3 °C min^−1^ from room temperature to 90 °C, then 1 °C min^−1^ from 90 °C to 100 °C.

### Preparation of PVP capped copper particles (PVP-Cu)

PVP (1.2 g, Mw = 10 000, TCI) was dissolved in 60 cm^3^ of 1,5-pentanediol (98%, Junsei), followed by addition of 1.2 g CuO (agglomerates, a few hundred nanometers to several micro-meters, Nissin Chemco, Japan). The mixture was heated to 80 °C with a stirring speed of 800 rpm. Hydrazine monohydrate (Junsei) was added into the mixture dropwise. This reaction was continued for 4 h. PVP-Cu particles were obtained by centrifugation with 1-propanol and drying under nitrogen.

### Characterization

Scanning electron microscopy (SEM) images of the commercial copper particles, PVP-Cu particles and Cu films were obtained using a JEOL JSM-6701F field-emission-type SEM. The thicknesses of the copper films were estimated from the cross-sectional SEM images for calculating the resistivities of the copper films. X-ray diffraction (XRD) patterns were collected using a Rigaku Miniflex-II diffractometer. Thermogravimetric differential thermal analysis (TG-DTA) was performed using a Shimadzu DTG-60H to study the decomposition of the mixture of CuA, IPA and ethanol as well as the remaining amount of organics in the sintered copper films. A four-point probe method using a Mitsubishi Chemical Analytech Loresta-GP with an ASP probe was used for measuring the sheet resistances of the copper films.

## Results and Discussion

### Influence of copper sources on the sintered copper film: Structure, sintering temperature, and resistivity

In this work, we primarily studied the decomposition process of the ink during the heating and the effect of residual organics on the resistivity. Before studying the above, we first studied the copper source for realizing excellent conductivity of the sintered film.

Here, CuA and CuF were selected as the copper sources for mixing with IPA and commercial copper particles (0.5 μm) to form copper inks. Our previous study showed that the decomposition of CuF with the help of IPA occurs at ~100 °C^24^. Besides CuF, CuA can also be used as a copper source in the inks. The decomposition of CuA occurs as shown in Eqs. 1 and 2,^29^,


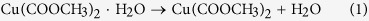



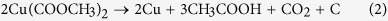


**Figure 1:**
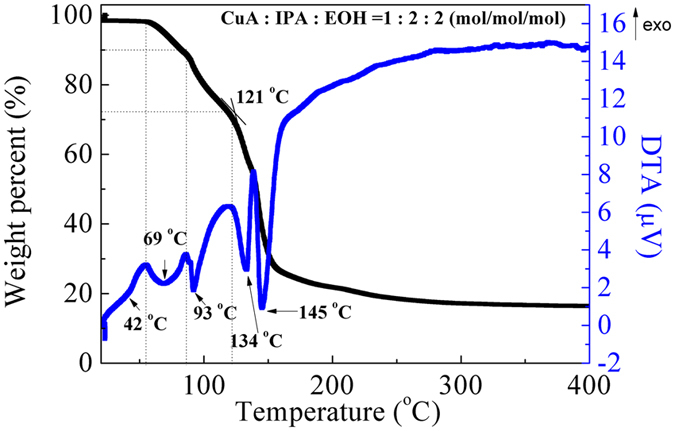
TG-DTA curves of mixture of CuA, IPA and ethanol (CuA/IPA/Ethanol = 1:2:2) under nitrogen. The sample was kept in the measurement equipment under nitrogen for 150 min for gas exchange to remove air. Then the sample was heated to 800 °C with a heating rate of 2.3 °C min^−1^.

To study the decomposition of the CuA complex with IPA in ethanol, the TG-DTA measurement was performed (Fig. 1). The weight loss at room temperature was due to the removal of a small quantity of ethanol and water because of gas exchange. The endothermic peaks at 42 °C and 69 °C mainly represent the evaporation of water from dehydration of CuA (9 wt% weight losses, Eq. 1). The endothermic peak at 93 °C represents the evaporation of ethanol and small amount of IPA (~20 wt% weight loss). The endothermic peaks at 134 °C and 145 °C are corresponding to the decomposition of CuA. The decomposition of CuA occurred from 120 °C onward was complete at ~300 °C.

**Figure 2:**
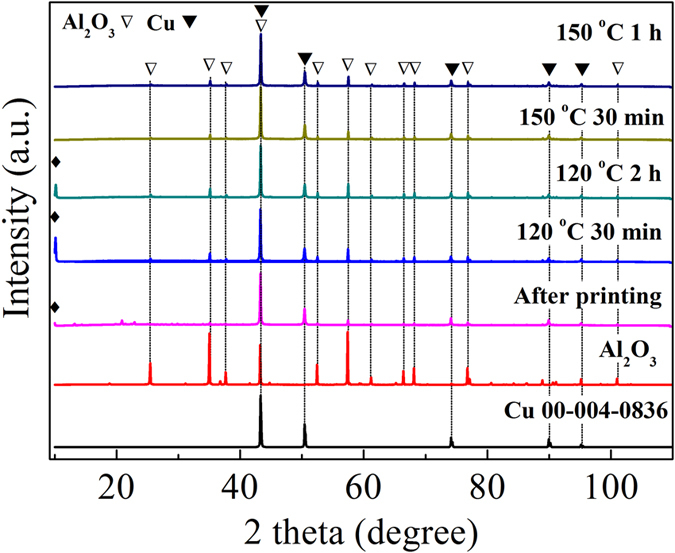
XRD patterns of Cu/CuA-IPA-EtOH inks after printing and sintering at 120 °C and 150 °C under nitrogen for various time. An impurity peak which is related to the printed ink at 10° (2*θ*) is marked with closed diamond. Peaks from the Cu layer and Al_2_O_3_ substrate are labelled with closed and open triangles, respectively.

**Figure 3:**
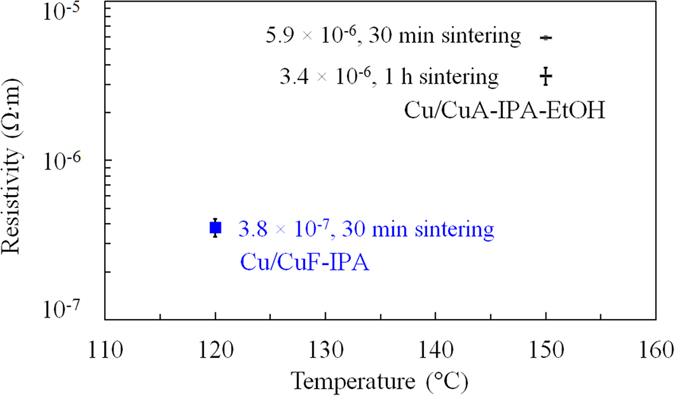
Resistivities of copper films sintered using Cu/CuF-IPA and Cu/CuA-IPA-EtOH inks at various temperatures. Error bars show the standard deviation of the resistivity for each sample.

Based on the above TG results, the prepared Cu/CuA-IPA-EtOH inks were sintered at 120 °C under nitrogen for various times. After sintering for 30 min and 2 h at 120 °C, there was an impurity peak at ~10° (2θ) in the XRD patterns for both the sintered films (Fig. 2). This impurity peak was the same as the peak from the printed films, indicating that the inks did not decompose completely at 120 °C for 30 min and 2 h. To obtain pure copper films, the sintering temperature was increased to 150 °C. Following sintering for 30 min and 1 h, the impurity peak at ~10° (2θ) disappeared (Fig. 2). There were only metallic copper peaks in the sintered copper films (Fig. 2). The resistivities of the films following sintering for 30 min and 1 h at 150 °C under nitrogen were 5.9 × 10^−6^ and 3.4 × 10^−6^ Ω·m, respectively (Fig. 3).

According to the TG/DTA curve in our previous study, the decomposition of CuF-IPA complex can happen at lower temperature (~100 °C)^24^. In addition, the resistivity of the film formed using the Cu/CuF-IPA inks after sintering at 120 °C for 30 min was 3.8 × 10^−7^ Ω·m (Fig. 3). Lower resistivity and sintering temperature were achieved using the Cu/CuF-IPA inks compared to the Cu/CuA-IPA inks, which is due to the effect of carbon from the decomposition of CuA (Fig. S2). Hence, CuF was chosen as the copper source for the following study.

### Effect of copper particle size on the resistivity of sintered copper film

After choosing the copper source, we studied the decomposition process of the ink during the heating process by changing the size of the added particles.

**Figure 4:**
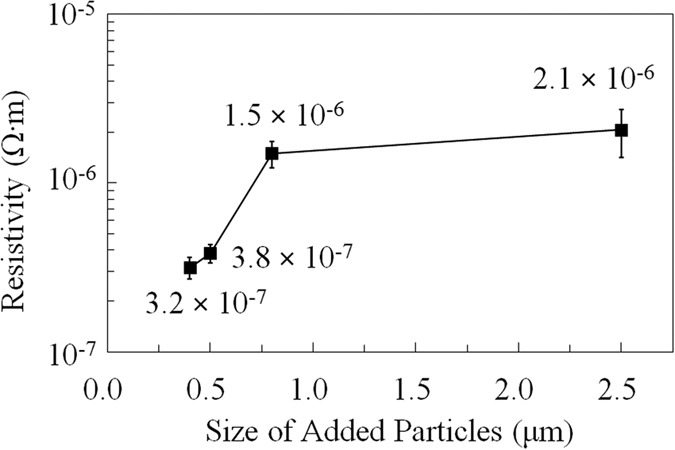
Resistivities of the copper films obtained after sintering at 120 °C using Cu/CuF-IPA inks inks with the added copper particles of various sizes: 0.4, 0.5, 0.8, and 2.5 μm. Error bars show the standard deviation of the resistivity for each sample.

**Figure 5:**
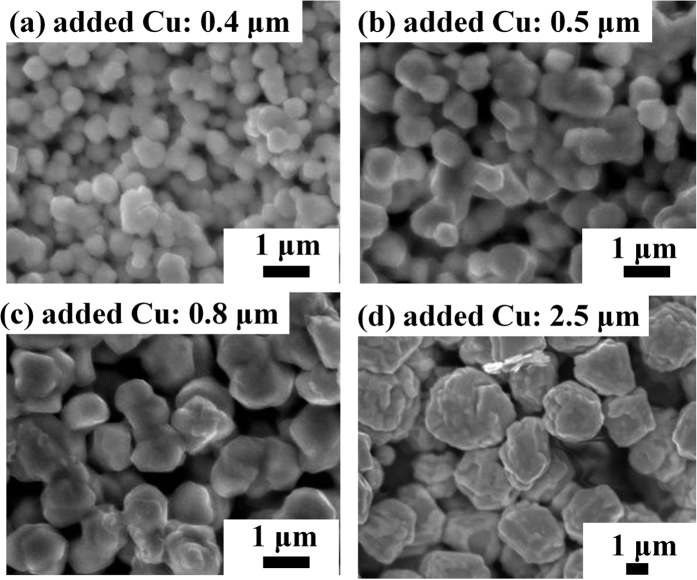
SEM images of the copper films obtained after sintering at 120 °C using Cu/CuF-IPA inks with the added copper particles of various sizes: (**a**) 0.4, (**b**) 0.5, (**c**) 0.8, and (d) 2.5 μm.

Previously, we proposed a novel method of mixing the copper complex and added copper particles for achieving low resistivity by sintering at a low temperature^24^. However, the decomposition process of the ink is still unclear. In this work, we investigated this process by adding copper particles of various sizes, 0.4, 0.5, 0.8 and 2.5 μm. All added commercial copper particles used have wide size distribution (Fig. S1, 0.4 ± 0.1, 0.5 ± 0.2, 0.8 ± 0.2, and 2.5 ± 0.7 μm) and bigger sized samples show wider size distributions. The wide size distributions of added copper particles can help improve the packing density of the sintered films. However, we will see in the following resistivity results that the films made from bigger copper particles have higher resistivities, which indicates that it is not the size distribution of the added copper particles but other factor having significant influence to the film resistivity. The resistivities of the obtained copper films after sintering at 120 °C under nitrogen for 30 min are given in Fig. 4. The copper films prepared using copper particles of size 0.4, 0.5, 0.8, and 2.5 μm showed resistivities of 3.2 × 10^−7^, 3.8 × 10^−7^, 1.5 × 10^−6^, and 2.1 × 10^−6^ Ω·m, respectively. According to the Gibbs-Thomson equation^30^, which expresses the relationship between the particle size and the melting point, micron-sized particles (Fig. S1) cannot melt at such a low sintering temperature, and they retain their original sizes and shapes. The low resistivities of the sintered copper films can be ascribed to the contact between particles. The resistivity of the film became lower with a decrease in the size of the added copper particles. This is because the smaller the particles, the smaller the remaining vacancies are. Under the same experiment conditions (oven, sintering temperature and time, heating profile) and ink compositions, all samples can be considered having the same evaporation rate of IPA, thereby IPA evaporation rate is not the reason causing the significant difference in the resistivity of the sintered copper films. As reported for the sintering of copper particles/copper complex ink, the copper from the complex was generated and grew on the surface of added copper particles to form the final copper film^24^,^31^. Low temperature sintering of copper particles without the metal complexes was also examined. No conductive copper layer was obtained in this case. Moreover, the copper layer was very fragile and many particles detached from the alumina substrate as indicated in Fig. S3. The results clearly indicate that the copper complexes are indispensable for the preparation of conductive stable copper layers. Copper atoms generated from CuF can fill the vacancy between the added copper particles by deposition. This result is consistent with the morphologies of the sintered films (Fig. 5). More connections among copper particles were observed in the sintered films with smaller added copper particles. The connections among particles may increase the percolation paths of electrons, and thus, decrease the resistivity of the sintered copper films. By using 0.4 μm copper particles in the inks, the lowest resistivity of the films sintered at 120 °C was 19 times that of bulk copper. For the same molar ratio of CuF to IPA and the same amount of added copper particles, the vacancy (space) among the added copper particles with a larger size is bigger than that among added copper particles of smaller sizes. Therefore, the deposition of copper atoms generated from CuF to bridge the gaps or fill the voids among the added copper particles becomes more effective when the size of the added copper particles decreases. Below a certain size of the added copper particles (i.e., 0.8 μm), the copper atoms generated from CuF to fill the space among the added copper particles strongly correlate with the decrease in the resistivity of the film, and we could observe a linear relationship between the size of the added copper particles and the resistivity. Above this size, the generated copper atoms may be insufficient for making connections and filling the space among the added copper particles. Hence, the relationship between the resistivity and the size of the added copper particles is nonlinear when copper particles with a large size are used (Fig. 4).

### Effect of organic residues on the resistivity of sintered copper film

According to the above results, the smaller the added copper particles are, the lower the resistivity is because there are fewer vacancies because of copper atom deposition from CuF. We then prepared smaller particles using PVP as the capping agent to replace the added commercial copper particles for the preparation of inks and sintered the ink at a lower temperature (100 °C).

**Figure 6:**
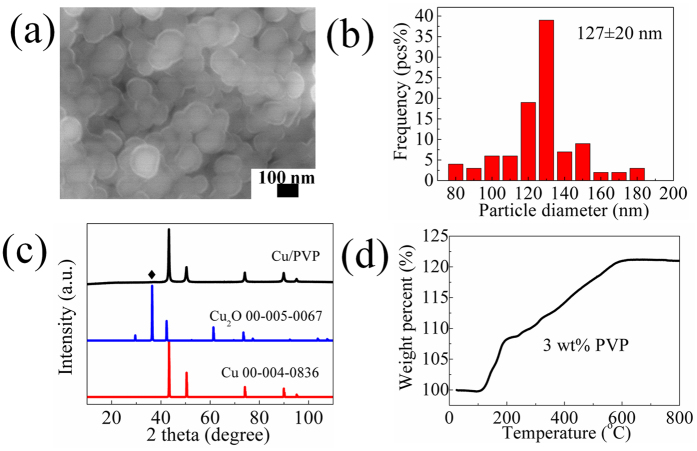
SEM image (**a**), size distribution (**b**), XRD pattern (**c**), and TG (**d**) of the synthesized PVP-Cu particles.

PVP-Cu particles of 127 ± 20 nm (Fig. 6a and b) showed a minor peak of Cu_2_O, besides the main peaks from metallic copper, in the XRD pattern (Fig. 6c). The weight percent of PVP in the PVP-Cu particles was 3 wt%, determined from the TGA result of the PVP-Cu particles (it was calculated from the amount of generated CuO after oxidation) (Fig. 6d). After sintering for 1 h and 2 h, metallic copper films without any oxides were obtained (Fig. S4). This can be attributed to the role of IPA and the generated hydrogen, which can convert copper oxide to copper and maintain a reducing atmosphere, respectively.

**Figure 7:**
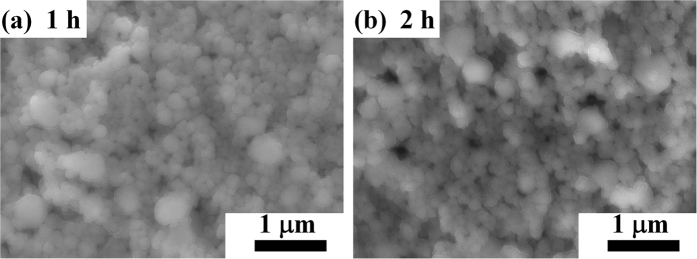
SEM images of copper films obtained using PVP-Cu/CuF-IPA inks after sintering at 100 °C for 1 and 2 h. (CuF/IPA = 1: 2 (mol/mol), 50 wt% Cu).

Figure 7 shows the SEM images of the copper films obtained using PVP-Cu/CuF-IPA inks after sintering at 100 °C for 1 and 2 h. From the SEM images, it was observed that the smaller PVP-Cu particles, added as copper particles to replace commercial particles for the preparation of ink, exhibit fewer vacancies. This result is consistent with our assumptions and the above results. However, the conductivity of the sintered films did not show better values.

Table 1 shows the resistivities of copper films obtained using PVP-Cu/CuF-IPA inks after sintering at 100 °C for 1 and 2 h. After sintering for 2 h, the film had a resistivity of 1 × 10^−4^ Ω·m. Upon decreasing the sintering time to 1 h, the resistivity of the copper film was beyond the range of measurement. In contrast, the copper film sintered at 100 °C for 1 h using the ink composed of 0.4 μm commercial copper particles (CuF/IPA = 1:2 (mol/mol), 50 wt% Cu) shows some conductivity (sheet resistance of 4.3 × 10^−2^ Ω/sq).

**Figure 8:**
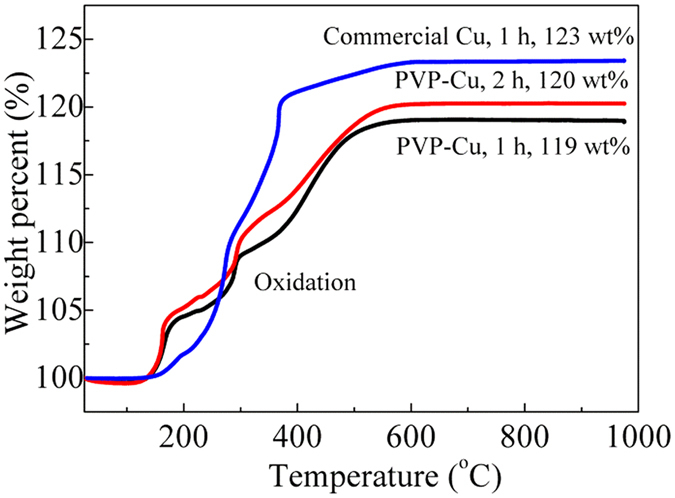
TGA curves of copper films obtained using Cu (commercially available, 0.4 μmφ)/CuF-IPA inks after sintering at 100 °C for 1 h (blue) and PVP-Cu/CuF-IPA inks after sintering at 100 °C for 1 (black) and 2 h (red). (CuF/IPA = 1:2 (mol/mol), 50 wt% Cu).

**Figure 9:**
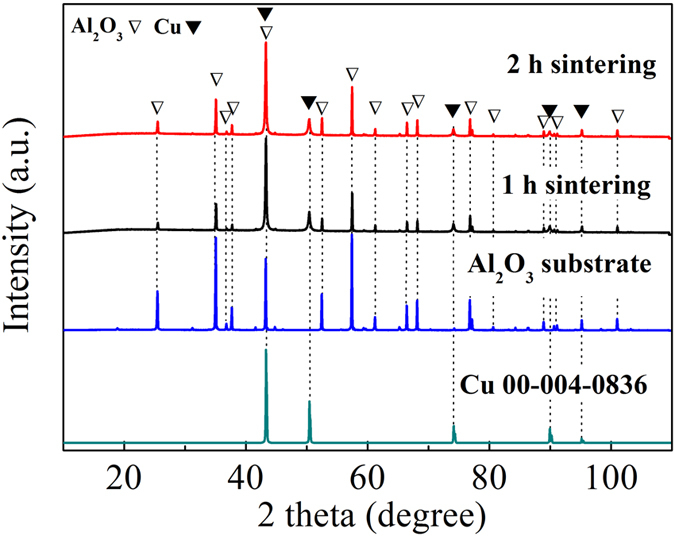
XRD patterns of copper films obtained using PVP-Cu/CuF-IPA inks after sintering at 100 °C for 1 and 2 h (CuF/IPA = 1:1 (mol/mol), 30 wt% PVP-Cu). Peaks from Cu and Al_2_O_3_ substrate are labelled with closed and open triangles, respectively.

To study the underlying reason, TG analyses were perfomed. Figure 8 shows the TGA curves of copper films obtained using Cu (0.4 μmϕ)/CuF-IPA inks after sintering at 100 °C for 1 h and PVP-Cu/CuF-IPA inks after sintering at 100 °C for 1 and 2 h. The amounts of organic residues were 2 wt% and 5 wt% in the copper films after sintering using Cu (0.4 μmϕ)/CuF-IPA inks and PVP-Cu/CuF-IPA inks after sintering at 100 °C for 1 h, respectively. In combination with the SEM results (Figs 5a and 7a), it can be deduced that a greater amount of organic residues in the copper film formed with the PVP-Cu/CuF-IPA ink after sintering at 100 °C for 1 h led to its high resistivity. In addition, using PVP-Cu/CuF-IPA inks, an increase in the sintering time from 1 h to 2 h resulted in a decrease in the amount of organics in the copper film after sintering at 100 °C from 5 wt% to 4 wt%, and a decrease in the film resistivity. Therefore, in order to further reduce the resistivity of the sintered copper film, the amounts of PVP-Cu and IPA were reduced. The mole ratio of CuF to IPA of 1:2 was changed to 1:1. The amount of added PVP-Cu particles was reduced from 50 wt% to 30 wt% to reduce the amount of PVP in the prepared ink. Following sintering at 100 °C for 2 h using this ink, the pure copper film (Fig. 9) can achieve a resistivity of 7 × 10^−6^ Ω·m (Table 1).

Based on the above results, it can be concluded that by using the added copper particles with the same capping agent, the resistivity of the conductive film obtained using the mixture of copper complex and the added copper particles as the MOD ink depends on the size of the added copper particle. The smaller the added copper particles, the lower is the resistivity. When the size of the added copper particles increases beyond critical value, the number of generated copper atoms may not be enough to make connections and fill the space among the added copper particles, and thus, the resistivity cannot decrease further. When the added copper particles with different capping agents are used for preparing the MOD ink, the amount of organics plays a critical role in reducing the resistivity of the obtained copper film when the degree of vacancies is similar. These results also indicate that the method of using a CuF-IPA complex mixed with copper particles for the preparation of copper-based MOD inks has a general applicability for low-temperature sintering of conductive copper films. Not only commercial copper particles but also synthesized PVP-Cu can be used as the added copper particles in the dispersions.

## Conclusions

In summary, copper-based MOD inks composed of a mixture of copper complex and copper particles are favourable for achieving low resistivity of copper films (10^−7^–10^−6^ Ω·m order of magnitude) at a low sintering temperature (less than 120 °C) under nitrogen gas. The choice of copper salts for the copper complexes and the influence of the size of the added commercial copper particles on the resistivity of the sintered copper films were systematically studied. In addition, smaller PVP-Cu particles were also used to replace commercial copper particles in the inks and demonstrate the general applicability of this method. It was found that CuF is better than CuA as the copper source for producing a sintered copper film with lower resistivity at a lower sintering temperature. Using CuF, a resistivity of 3.8 × 10^−7^ Ω·m was achieved for the copper film after sintering at 120 °C for 30 min. The resistivity of the sintered copper films decreased with a decrease in the size of the added copper particles. The lowest resistivity of 3.2 × 10^−7^ Ω·m was obtained using 0.4 μm commercial copper particles at a sintering temperature of 120 °C for 30 min. In addition, using PVP-Cu particles (127 ± 20 nm) instead of the commercial particles for preparing copper-based MOD dispersions also resulted in a copper film with a low resistivity (7 × 10^−6^ Ω·m) at a low sintering temperature of 100 °C. The above results demonstrate the effectiveness of using mixtures of copper complex and added copper particles as the dispersions for achieving low resistivity (10^−7^–10^−6^ Ω·m order of magnitude) at a low sintering temperature (100 °C–120 °C) without using any reductive gas.

## Additional Information

**How to cite this article**: Yong, Y. *et al*. Effect of decomposition and organic residues on resistivity of copper films fabricated via low-temperature sintering of complex particle mixed dispersions. *Sci. Rep.* **7**, 45150; doi: 10.1038/srep45150 (2017).

**Publisher's note:** Springer Nature remains neutral with regard to jurisdictional claims in published maps and institutional affiliations.

## Supplementary Material

Supporting Information

## Figures and Tables

**Table 1:** Resistivities of the obtained copper films using PVP-Cu/CuF-IPA inks following sintering at 100 °C under nitrogen.

Sintering time at 100 °C	Resistivity (Ω m)	
	CuF/IPA = 1:2 50 wt% PVP-Cu	CuF/IPA = 1:1 30 wt% PVP-Cu
1 h	Over range (5.6 μm thickness)	19.4 (4.5 μm thickness)
2 h	1 × 10^−4^ (10.8 μm thickness)	7 × 10^−6^ (3.9 μm thickness)

(CuF/IPA = 1:2, 50 wt% PVP-Cu and CuF/IPA = 1:1 (mol/mol), 30 wt% PVP-Cu).
