# Supplementary material for: Effect of decomposition and organic residues on resistivity of copper films fabricated via low-temperature sintering of complex particle mixed dispersions
Source: Sci Rep. 2017 Mar 24;7:45150. doi: 10.1038/srep45150 (PMC5364527; doi:10.1038/srep45150)
Supplement: Supporting Information [file srep45150-s1.doc]

**Supporting Information**

**Effect of decomposition and organic residues on resistivity of copper films fabricated via low-temperature sintering of complex particle mixed dispersions**

Yingqiong Yong,^a^ Mai Thanh Nguyen,^a^ Hiroki Tsukamoto,^a^ Masaki Matsubara,^a,b^ and Ying-Chih Liao^a,c^, Tetsu Yonezawa^a^,*

^a^Division of Materials Science and Engineering, Faculty of Engineering, Hokkaido University, Kita 13 Nishi 8, Kita-ku, Sapporo, Hokkaido 060-8628, Japan

^b^Department of Materials and Environment Engineering, National Institute of Technology, Sendai College, 48 Nodayama, Medeshima-Shiote, Natori-shi, Miyagi 981-1239, Japan

^c^Department of Chemical Engineering, Faculty of Engineering, National Taiwan University, No. 1, Section 4, Roosevelt Rd., Da’an District, Taipei, 10617 Taiwan
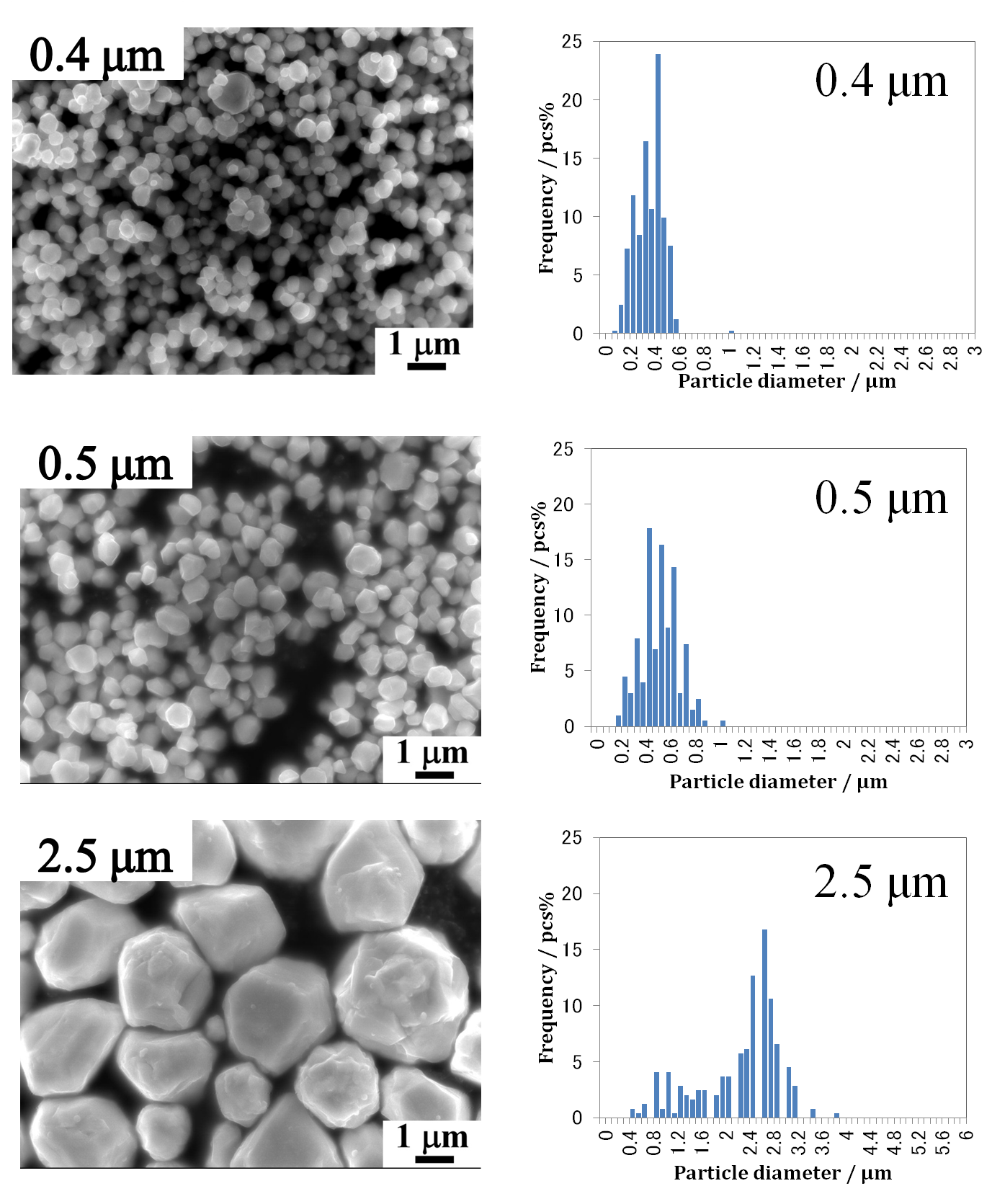


**Figure S1.** SEM images and size distributions of added commercially available (DOWA) copper particles. The copper particles with size of 0.8 μm can be seen in supporting information of our previous study.^23^


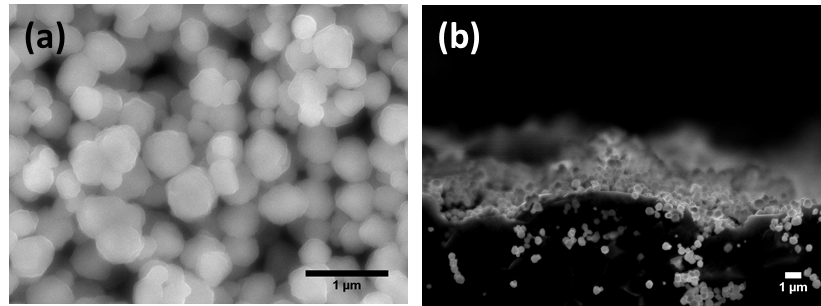


**Figure S2.** Surface (a) and cross-sectional (b) SEM images of sintered commercial copper fine particles (size = 0.4 μm). Comparing the SEM image in Figure S1, independent particles without any changes can also be observed in (a). No necking or connecting of particles can be observed. In (b), no clear connection among the particles can be observed and some particles were detached from the substrate during cutting.

**
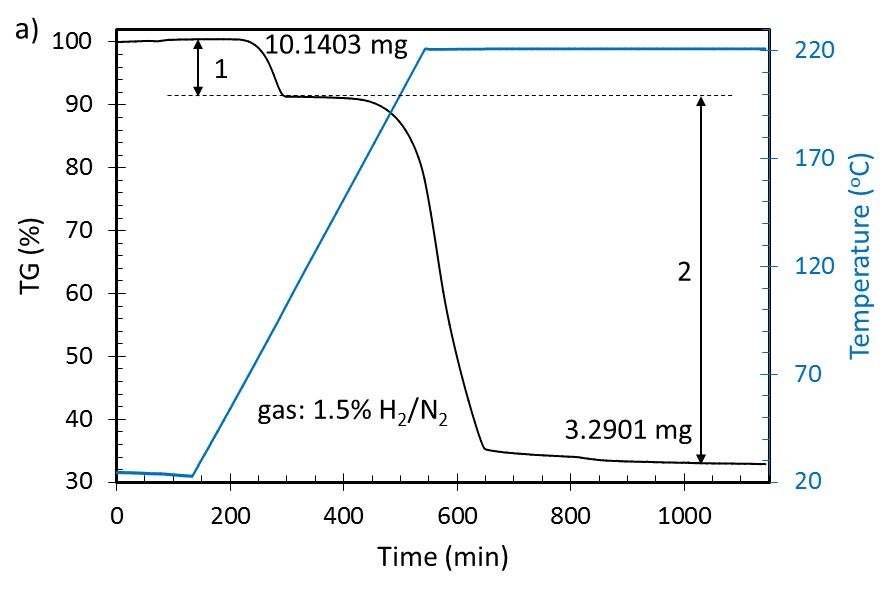

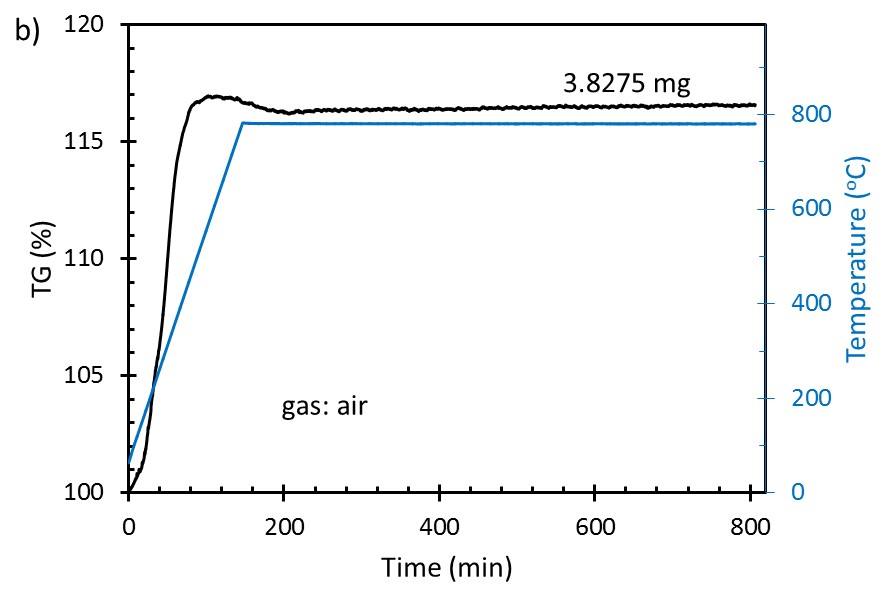
**

**Figure S3.** (a) TG curve of copper acetate monohydrate (CuA) under 1.5% H_2_/N_2_ gas flow (flow rate: 50 cm^3^ min^-1^, heating rate: 0.5 °C min^-1^). (b) TG curve of the solid products obtained after (a) via heating under air flow up to 800 °C (flow rate: 50 cm^3^ min^-1^, heating rate: 5 °C min^-1^).

1.5% H_2_/N_2_ was chosen to prevent the oxidation of Cu and higher temperature (i.e. 220 °C) in order to understand the decomposition behavior of copper acetate (the decomposition starts around 150 °C).

Figure S2(a): **Step 1** with 9.0 % mass loss corresponds to the release of water in copper acetate monohydrate (Equation 1: Cu(CH_3_COO)_2·_H_2_O → Cu(CH_3_COO)_2_*(s)* + H_2_O*(g)*, main text), and **step 2** with 58 % mass loss corresponds to the gas generated in the decomposition of copper acetate. In addition, solid products (33 %) comprise of Cu and C.

Figure S2(b): The complete removal of C (to form CO_2_) and oxidation of Cu occurred by heating under air. From the final weight of CuO, the amount of Cu in the solid products of the TG sample in (a) can be estimated. It shows 31 wt% Cu and 2 wt% C left in the solid products (total solid products: 33wt% of initial CuA). These results are in good agreement with the theoretical estimation of the weight loss based on Equation 2 shown in main text: 2Cu(CH_3_COO)_2_ → 2Cu*(s)* + 3CH_3_COOH*(g)* + CO_2_*(g)* + C*(s)*.


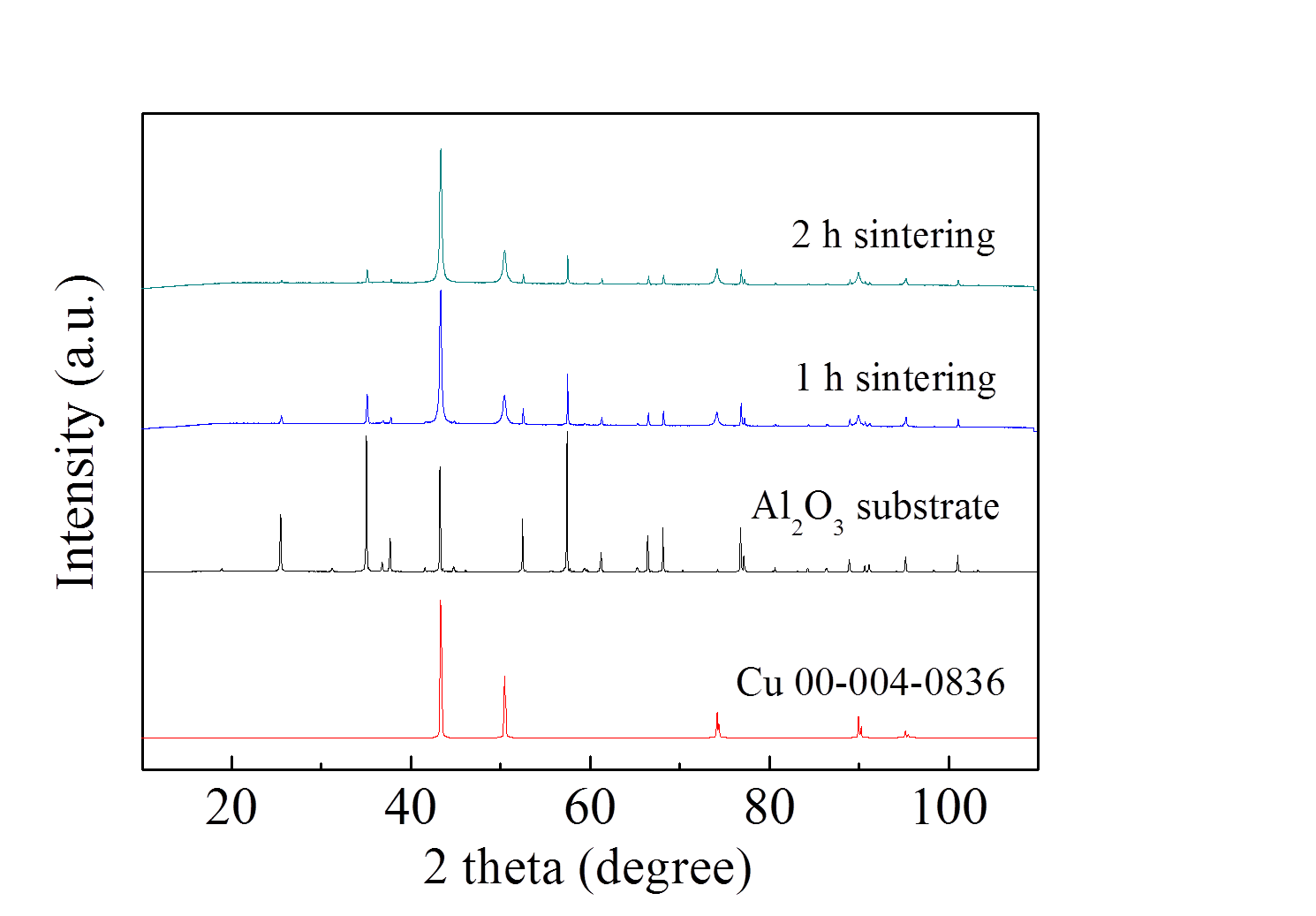


**Figure S4.** XRD patterns of copper films obtained using PVP-Cu/CuF-IPA inks (CuF : IPA = 1 : 2 (mol/mol), 50 wt% PVP-Cu) after sintering at 100 °C for 1 and 2 h.
